# Alginate dressings for treating pressure ulcers

**DOI:** 10.1590/1516-3180.20151335T2

**Published:** 2015-08-21

**Authors:** Jo C. Dumville, Samantha J. Keogh, Zhenmi Liu, Nikki Stubbs, Rachel M. Walker, Mathew Fortnam

## Abstract

**BACKGROUND:**

Pressure ulcers, also known as bedsores, decubitus ulcers and pressure injuries, are localised areas of injury to the skin or the underlying tissue, or both. Dressings are widely used to treat pressure ulcers and there are many options to choose from including alginate dressings. A clear and current overview of current evidence is required to facilitate decision-making regarding dressing use for the treatment of pressure ulcers. This review is part of a suite of Cochrane reviews investigating the use of dressings in the treatment of pressure ulcers. Each review will focus on a particular dressing type.

**OBJECTIVES:**

To assess the effects of alginate dressings for treating pressure ulcers in any care setting.

**METHODS:**

*Search methods: * For this review, in April 2015 we searched the following databases the Cochrane Wounds Group Specialised Register; The Cochrane Central Register of Controlled Trials (CENTRAL) (The Cochrane Library); Ovid MEDLINE; Ovid MEDLINE (In-Process & Other Non-Indexed Citations); Ovid EMBASE; and EBSCO CINAHL. There were no restrictions based on language or date of publication.

*Selection criteria: *Published or unpublished randomised controlled trials (RCTs) comparing the effects of alginate with alternative wound dressings or no dressing in the treatment of pressure ulcers (stage II or above).

*Data collection and analysis: *Two review authors independently performed study selection, risk of bias assessment and data extraction.

**MAIN RESULTS:**

We included six studies (336 participants) in this review; all studies had two arms. The included studies compared alginate dressings with six other interventions that included: hydrocolloid dressings, silver containing alginate dressings, and radiant heat therapy. Each of the six comparisons included just one study and these had limited participant numbers and short follow-up times. All the evidence was of low or very low quality. Where data were available there was no evidence of a difference between alginate dressings and alternative treatments in terms of complete wound healing or adverse events.

**AUTHORS' CONCLUSIONS:**

The relative effects of alginate dressings compared with alternative treatments are unclear. The existing trials are small, of short duration and at risk of bias. Decision makers may wish to consider aspects such as cost of dressings and the wound management properties offered by each dressing type, for example, exudate management.

**The abstract of this review is available from:** http://onlinelibrary.wiley.com/doi/10.1002/14651858.CD011277.pub2/abstract
